# Relation of Resting Membrane Polarization and Insulin Resistance in Critically Ill Patients

**DOI:** 10.1186/2197-425X-3-S1-A520

**Published:** 2015-10-01

**Authors:** S Koch, T Wollersheim, K Mai, K Haas, C Spies, J Grosskreutz, S Weber-Carstens

**Affiliations:** Charité - Universitätsmedizin Berlin, Department of Anesthesiology and Intensive Care Medicine, Berlin, Germany; Charité - Universitätsmedizin Berlin, Department of Endocrinology, Diabetes and Nutrition, Berlin, Germany; Univerisity of Jena, Department of Neurology, Jena, Germany

## Introduction

Critically ill patients feature depolarization of the resting membrane potential and reduced membrane excitability in motor nerve and muscle [1, 2], which is correlated to ICU-acquired weakness and an increased insulin resistance [3].

## Objectives

Since insulin is one agonist of the Na-K-pump, controlling resting membrane potential in muscle and nerve, we hypothesized that insulin resistance is linked to motor nerve resting membrane depolarisation in critically ill patients.

## Methods

We recorded compound motor action potential from the abductor pollicis brevis muscle in ICU patients to test excitability measures of the median-nerve at baseline and during euglycemic-hyperinsulinemic clamp, proving resting membrane polarization. the recovery-of-excitability following a supra-maximal conditioning stimulus was tested at 18 conditioning test intervals, decreasing from 200 to 2 ms in geometric progression.

Insulin sensitivity index (ISI), as marker of myocellular insulin resistance, was calculated during steady state condition of euglycemic-hyperinsulinemic clamp.

## Results

**Figure 1:**
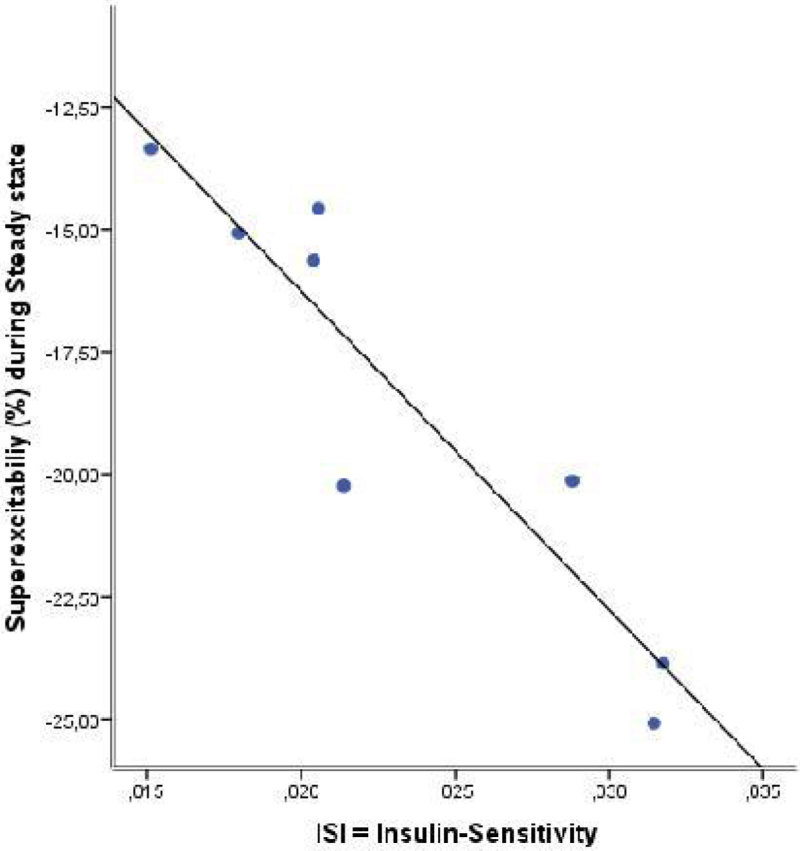
Correlation between resting membrane polarisation and insulin resistance (ISI = Insulin sensitivity index; indicates ratio of glucose infusion rate to the serum insulin concentration during steady state). Less negative Superexcitability indicates resting membrane depolarization; lower ISI values indicate higher insulin resistance.

10 ICU patients and 31 healthy controls were enrolled in this trial. Compared to control group, ICU-patients exhibited depolarization of resting membrane potential (superexcitability in healthy controls -25 + 6.1% versus -18.5 + 4.5% in ICU patients; p = 0.003). the resting membrane depolarization was significantly correlated to ISI (R² = 0.858; p = 0.003), where pronounced insulin resistance correlates with pronounced resting membrane depolarization (Figure 1), indicating that membrane repolarization after insulin stimulation of Na-K-pump is reduced in patients with severe insulin resistance. K+ plasma levels were not correlated with membrane depolarization.

## Conclusions

Resting membrane depolarization in critically ill patients is correlated to insulin resistance. Patients with severe insulin resistance reveal a failure of repolarization, so that high dosage of insulin administration does not facilitate rectification of membrane polarization.

## Grant Acknowledgment

DFG / DGAI supported this work.
